# Anti-lymphangiogenic properties of mTOR inhibitors in head and neck squamous cell carcinoma experimental models

**DOI:** 10.1186/1471-2407-13-320

**Published:** 2013-07-01

**Authors:** Oleksandr Ekshyyan, Tara N Moore-Medlin, Matthew C Raley, Kunal Sonavane, Xiaohua Rong, Michael A Brodt, Fleurette Abreo, Jonathan Steven Alexander, Cherie-Ann O Nathan

**Affiliations:** 1 Department of Otolaryngology/Head and Neck Surgery, Louisiana State University Health Sciences Center, Shreveport, LA, USA; 2 Feist-Weiller Cancer Center, LSUHSC, Shreveport, LA, USA; 3 Department of Pathology, LSUHSC, Shreveport, LA, USA; 4 Department of Cellular and Molecular Physiology, LSUHSC, Shreveport, LA, USA

**Keywords:** Head and neck squamous cell carcinoma, Rapamycin, mTOR inhibitors, mTOR, VEGFR-3, VEGFR-2, Lymph node metastasis

## Abstract

**Background:**

Tumor dissemination to cervical lymph nodes via lymphatics represents the first step in the metastasis of head and neck squamous cell carcinoma (HNSCC) and is the most significant predictor of tumor recurrence decreasing survival by 50%. The lymphatic suppressing properties of mTOR inhibitors are not yet well understood.

**Methods:**

Lymphatic inhibiting effects of rapamycin were evaluated *in vitro* using two lymphatic endothelial cell (LEC) lines. An orthotopic mouse model of HNSCC (OSC-19 cells) was used to evaluate anti-lymphangiogenic effects of rapamycin *in vivo*. The incidence of cervical lymph node metastases, numbers of tumor-free lymphatic vessels and those invaded by tumor cells in mouse lingual tissue, and expression of pro-lymphangiogenic markers were assessed.

**Results:**

Rapamycin significantly decreased lymphatic vascular density (*p* = 0.027), reduced the fraction of lymphatic vessels invaded by tumor cells in tongue tissue (*p* = 0.013) and decreased metastasis-positive lymph nodes (*p* = 0.04). Rapamycin also significantly attenuated the extent of metastatic tumor cell spread within lymph nodes (*p* < 0.0001). We found that rapamycin significantly reduced LEC proliferation and was correlated with decreased VEGFR-3 expression in both LEC, and in some HNSCC cell lines.

**Conclusions:**

The results of this study demonstrate anti-lymphangiogenic properties of mTOR inhibitors in HNSCC. mTOR inhibitors suppress autocrine and paracrine growth stimulation of tumor and lymphatic endothelial cells by impairing VEGF-C/VEGFR-3 axis and release of soluble VEGFR-2. In a murine HNSCC orthotopic model rapamycin significantly suppressed lymphovascular invasion, decreased cervical lymph node metastasis and delayed the spread of metastatic tumor cells within the lymph nodes.

## Background

Despite advances in treatment options, there have been no significant improvements in 5-year survival rates of head and neck squamous cell carcinoma (HNSCC) patients in the past four decades. While the 1-year survival rate is 81%, the 5-year survival rate remains at ~45% for all stages of oral cancer [1]. Metastasis to regional lymph nodes occurs in 30-40% of HNSCC cases [2], and is associated with poor prognosis and low survival [2,3]. Lymphatogenous spread of cancer cells is a significant problem in HNSCC reflecting the rich lymphatic supply in the head and neck. High risk features, such as lymphovascular invasion and extracapsular spread significantly increase the risk of both local recurrence, and distant metastasis. Consequently postoperative adjuvant chemoradiotherapy is recommended to decrease recurrence rates [4]. De Carvalho in a prospective analysis of 170 cases of previously untreated patients with laryngeal or hypopharyngeal squamous cell carcinoma found that macroscopic extracapsular tumor spread increased the risk of recurrence 3.5-fold compared with patients with no evidence of metastasis at their initial diagnosis, or patients in whom the tumor was confined to the lymph node [5]. In another study, patients with extracapsular nodal spread had significantly higher rates of recurrent disease and distant metastasis [6].

Tumor cell spread to regional lymph nodes through lymphatic vessels is known to be one of the worst prognostic factors, decreasing survival by 50%. Formation of new tumor-associated lymphatic vessels through lymphangiogenesis plays an active role in the initiation and progression of metastatic disease spread as demonstrated by the significant correlation between intratumoral lymphatic vascular density and lymph node metastasis.

HNSCC is characterized by persistent activation of the Akt/mTOR pathway that triggers a cascade of molecular events central to carcinogenesis including cancer cell survival, cell cycle progression, proliferation, transcription and translation, angiogenesis, invasion, and metastasis [7,8]. The Akt/mTOR pathway is a fundamental coordinator of several signaling pathways related to cell growth and division, and mTOR inhibitors effectively reduce proliferation in cells with constitutively upregulated Akt/mTOR signaling. The mammalian target of rapamycin (mTOR) signaling pathway is dysregulated in nearly all (99%) cases of HNSCC [9]. mTOR inhibitors depress translation of several mRNAs specifically required for tumor cell cycle progression, proliferation, and angiogenesis suppressing oncogenesis [10-15]. Because these pathways are commonly dysregulated in cancer, mTOR represents an attractive anti-tumor target. The mTOR inhibitor rapamycin (sirolimus) was approved by the FDA in 1999 to prevent renal transplant rejection [16] and is a clinically approved immunosuppressive agent with promising anti-tumor properties. Chronic use of rapamycin shows a good safety profile in renal transplantion [17,18] and is well tolerated with only mild and usually reversible side effects which include herpes simplex lesions, acne-like and maculopapular rash, and nail disorders. Dose-limiting toxicities consist of mucositis/stomatitis, asthenia, thrombocytopenia and hyperlipidemia [19].

Although the role of mTOR inhibitors is well established in renal cell carcinoma and recent phase 1 and 2 studies in solid tumors hold promise, their anti-lymphatic properties are not well characterized. Previously in collaboration with Dr. Silvio Gutkind’s group (Oral and Pharyngeal Cancer Branch, National Institute of Dental Research, NIH) using an orthotopic model of HNSCC generated by injection of UMSCC2 cells into the tongue of SCID/NOD mice we demonstrated significant inhibition of tumor growth, decreased lymphatic microvessel density and a decrease in the number of invaded lymph nodes after rapamycin and RAD001 treatment [20]. In the current study we expand the analysis of the anti-lymphatic properties of rapamycin by using an orthotopic murine model of HNSCC generated by injection of highly metastatic OSC-19 cells. Here we investigated the molecular mechanisms of rapalogue anti-lymphatic action and related anti-tumor effects.

## Methods

### Evaluation of the anti-lymphangiogenic effects of rapamycin in a regional metastasis model

All animal studies were carried out according to the protocol approved by the Louisiana State University Health Sciences Center Institutional Animal Care and Use Committee, in compliance with the Committee guidelines. Severe combined immunodeficient (SCID) male mice, 4 to 6 weeks of age (Charles River Laboratories, Wilmington, Massachusetts), were housed in a barrier facility and maintained on a normal diet ad libitum.

2 × 10^5^ OSC-19 cells, a highly invasive and metastasis-prone oral squamous carcinoma cell line, were injected into the basolateral region of the tongues of SCID mice. The mice were randomized into two groups (28 control mice and 25 rapamycin-treated mice ). 5 days after cell injections mice were given daily IP injections of vehicle (4% DMSO, 5.2% Tween 80, and 5.2% polyethylene glycol 400) or rapamycin at a dose of 5 mg/kg. 21 days after injection of OSC-19 cells mice were sacrificed. Lingual tissue and cervical lymph node samples were harvested. Mouse tongues were bisected and consecutive samples of lingual tissue and cervical lymph nodes were fixed in 10% neutral buffered formalin for 24 hours, processed and embedded in paraffin. Lingual tissue sections were stained with hematoxylin and eosin (H&E) and cross-sectional area of xenograft tumors was measured using Image-J software (NIH; Windows version). Cervical lymph node samples were examined microscopically by a pathologist using H&E and cytokeratin staining to determine the cervical lymph node metastasis incidence. The number of tumor-free lymphatic vessels and those invaded by tumor cells in mouse tongues was assessed by our pathologist using LYVE-1 (a lymphatic-specific biomarker) immunohistochemical staining (R&D Systems, Minneapolis, MN). Lymphatic vessels invaded by tumor cells were defined as those with the presence of tumor cells in the endothelium-lined space (i.e lymphovascular invasion). Blood microvascular density was assessed after immunohistochemical staining with CD31 (PECAM-1; Santa Cruz Biotechnology, Santa Cruz, CA). Individual microvessels were counted using a × 400 field (× 40 objective lens and × 10 ocular lens). At least three random fields within the tumor area were viewed and counted at × 400 magnification. Results were expressed as the average number of microvessels per field. Unpaired t test with Welch correction was used to evaluate the differences between treatment groups.

### Cell Lines

HMEC-1A cells are a human lymphatic endothelial cell line that was subcloned [21] from HMEC-1 cells – an immortalized cell culture, which is a combination of lymphatic and blood vascular endothelial cells [22]. HMEC-1A cells were maintained in MCDB 131 medium (Sigma-Aldrich), supplemented with 20mM HEPES, 1 ug/ml hydrocortisone, 10 ng/ml EGF and 10% fetal bovine serum (FBS).

SV-LEC cells, a stable mouse lymphatic endothelial cell line, was isolated from mesenteric adventitial tissue and shown to express specific lymphatic markers Prox-1, LYVE-1 and VEGFR-3 [23]. SV-LEC cells were cultured in DMEM/F12 medium supplemented with 10% FBS.

HNSCC cell line SCC40 (tongue cancer) was kindly provided by Dr. Susanne Gollin and PCI-15a (pyriform sinus cancer) was provided by Dr. Theresa L. Whiteside (both from the University of Pittsburgh Graduate School of Public Health). FaDu cells, established from hypopharyngeal SCC, were procured from ATCC. SCC40, PCI-15a and FaDu cultures were maintained in MEM media supplemented with 10% FBS and non-essential amino acids. 2 × 10^5^ OSC-19 cells, a gift from Dr. Eben L. Rosenthal (University of Alabama at Birmingham), were cultured in DMEM/F12 medium supplemented with 10% FBS.

### Cell Proliferation Assay

The effects of rapamycin (LC Laboratories, Woburn, MA) on proliferation of SV-LEC or HMEC-1A cells were determined by plating exponentially growing cells in 96-well plates (2,000 per well) with 200 μl of medium. The cells were incubated at 37°C for 3.5 hours for adherence and then treated with vehicle (DMSO) or various concentrations of rapamycin (1-1000 ng/ml) for time points ranging from 0 to 72 h. Cell proliferation was measured using a modified MTT (3-(4-5-dimethylthiazol-2-yl)-5-(3-carboxymethoxyphenyl)-2-(4-sulfophenyl)-2H-tetrazolium salt/phenazine methosulfate) system according to the manufacturer's instructions (CellTiter 96 AQueous cell proliferation assay; Promega Corp., Madison, WI).

### Detection of apoptosis in lymphatic endothelial cells by DAPI staining

SV-LEC (10,000 cells) or HMEC-1A (20,000 cells) were seeded on 12 mm circular glass cover slips in 24-well plates and allowed to attach for 4 h. Cells were then treated with 100 ng/ml of rapamycin or vehicle control (DMSO) for 72 h, washed with phosphate-buffered saline (PBS) and fixed in cold 2% paraformaldehyde for 15 min. Cells were then washed with PBS, fixed with cold 70% ethanol at -20°C for 1 h and stained with 1 mg/ml DAPI for 30 min in the dark. The coverslips were washed 2× with PBS, and mounted using DAKO fluorescent mounting fluid onto microscope slides. Cells were viewed and counted using a fluorescent Olympus Bx50 microscope using a 40× objective. The number of total and apoptotic cells were counted at least in four fields of each slide.

### Western Blot Analysis

Soluble proteins were extracted as previously described [24]. 30 ug of protein was loaded per well and the expression of tumor and lymphatic biomarkers evaluated by western blotting using the following antibodies: 4EBP1 (1:300 dilution), phospho-4EBP1 (serine 37/46; 1:300 dilution), total and phospho-S6 ribosomal protein (serine 235/236, 1:100), actin (1:3,000; – all above - Cell Signaling, Beverly, MA). VEGFR-3/Flt-4 antibody was used at a 1:100 dilution (R&D Systems, Minneapolis, MN). The expression levels of each marker were quantified after normalizing to actin scan density by immunoblotting.

### Vascular endothelial growth factor receptor-2 ELISA assay

The effects of rapamycin treatment on serum levels of soluble VEGFR-2 in mouse serum samples were determined using a mouse VEGFR-2 ELISA kit (R&D Systems, Minneapolis, MN) according to manufacturer's instructions.

## Results

### Anti-lymphatic effects of rapamycin in orthotopic HNSCC model

**Figure 1:**
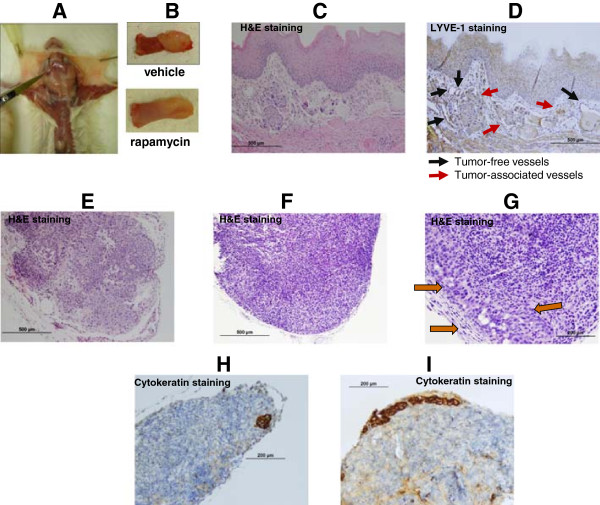
**Anti-lymphatic effects of rapamycin in the orthotopic OSCC tongue tumor model. A**, Extraction of cervical lymph nodes. **B**, Mouse tongue with OSC-19 tumors formed after tumor cells injection. **C-D**, Effects of rapamycin on the number of LYVE + lymphatic vessels invaded by tumor cells in mouse lingual tissues. **E-G**, Extent of lymph node metastasis. **E**, Lymph node (**H&E** stain) with extensive tumor involvement (control mouse). **F**, Lymph node (H&E stain), no tumor metastasis (rapamycin-treated mouse). **G**, Lymph node (H&E stain) with subcapsular metastasis indicated by arrows (rapamycin-treated mouse). **H-I**, Detection of lymph node metastasis using cytokeratin staining.

Anti-lymphatic effects of rapamycin were evaluated in the orthotopic OSCC tongue tumor model (Figure 1). OSC-19 cells injected into tongues of SCID mice formed tumors (Figure 1A) in all mice and yielded a reproducibly high rate of regional metastases by week 3. Rapamycin significantly inhibited tumor growth as measured by tumor cross-sectional area at the end of experiment. The mean total cross-sectional area was 27.4 ± 13.4 mm^2^ in control mice which was decreased to 8.4 ± 6.7 mm^2^ in rapacymin-treated mice (p < 0.0001).

Rapamycin significantly decreased intratumoral lymphatic vascular density from 9.1 ± 4.10 in control mice to 5.8 ± 1.18 in rapamycin-treated mice (p = 0.027) as well as the fraction of lymphatic vessels (identified by LYVE-1 staining) invaded by tumor cells in primary OSC-19 tumors obtained from mouse lingual tissue (Figure 1D). The percentage of lymphatic vessels invaded by tumor cells decreased from 62.78 ± 15.13% in controls to 40.44 ± 20.67 in the rapamycin-treated mice (p = 0.013).

H&E and cytokeratin stained slides of the cervical lymph nodes were analyzed by the study pathologist to determine the presence of metastases and the extent of spread within the lymph nodes. Following rapamycin treatment we observed a significant decrease in the incidence of cervical lymph node metastases (p = 0.04; Fisher exact test of independence). In the control group, 42 of the 66 (63.6%) lymph nodes evaluated revealed metastatic tumors, while in the rapamycin-treated group only 31 of the 68 (45.6%) lymph nodes evaluated showed metastasized tumors. This shows that the incidence of cervical lymph node metastases decreased by almost one third after rapamycin treatment. Rapamycin also significantly reduced the extent of tumor spread within the lymph nodes. In the control group 33 of the 42 (78.6%) lymph nodes with metastatic tumor showed extensive lymph node involvement. By comparison, in the rapamycin-treated group only 8 of the 31 (25.8%) lymph nodes with metastatic tumor showed extensive lymph node involvement, while 74.2% of the metastatic lymph nodes had only minimal tumor involvement that was localized to the subcapsular sinuses (p = 0.0001; Fisher exact test of independence; Figure 1E-I and Table 1).

**Table 1:** Cervical lymph node metastasis in orthotopic HNSCC mouse model

	**Control**	**Rapamycin**
**Number of positive cervical lymph nodes**	42/66 (63.6%)	31/68 (45.6%) *
**Extent of tumor cell spread within positive lymph nodes:**		
Extensive spread	33/42 (78.6%)	8/31 (25.8%) ****
Subcapsular	9/42 (21.4%)	23/31 (74.2%) ****

* - p < 0.05 (Fisher’s test).

**** - p < 0.0001 (Fisher’s test).

**Figure 2:**
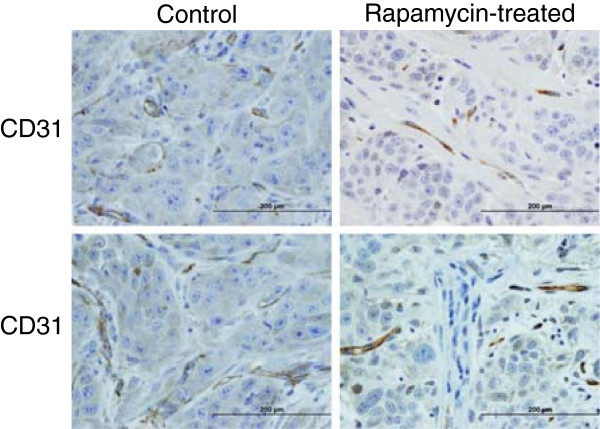
**Rapamycin reduces intratumoral blood microvessel density in the orthotopic OSCC tongue tumor model.** Representative CD31-stained sections from control and rapamycin treatment groups are shown.

We also assessed the effects of rapamycin on angiogenesis by quantitating the number of blood microvessels in CD31-stained sections of lingual tumor tissue (Figure 2). At × 400 magnification, the average blood vessel counts per field (mean ± SD) were: 23.36 ± 5.56 blood microvessels in control tumors compared to 14.94 ± 3.79 for rapamycin-treated tumors (p < 0.0001; unpaired t test with Welch correction). This shows a significant 36% reduction in blood vessel density following rapamycin treatment.

**Figure 3:**
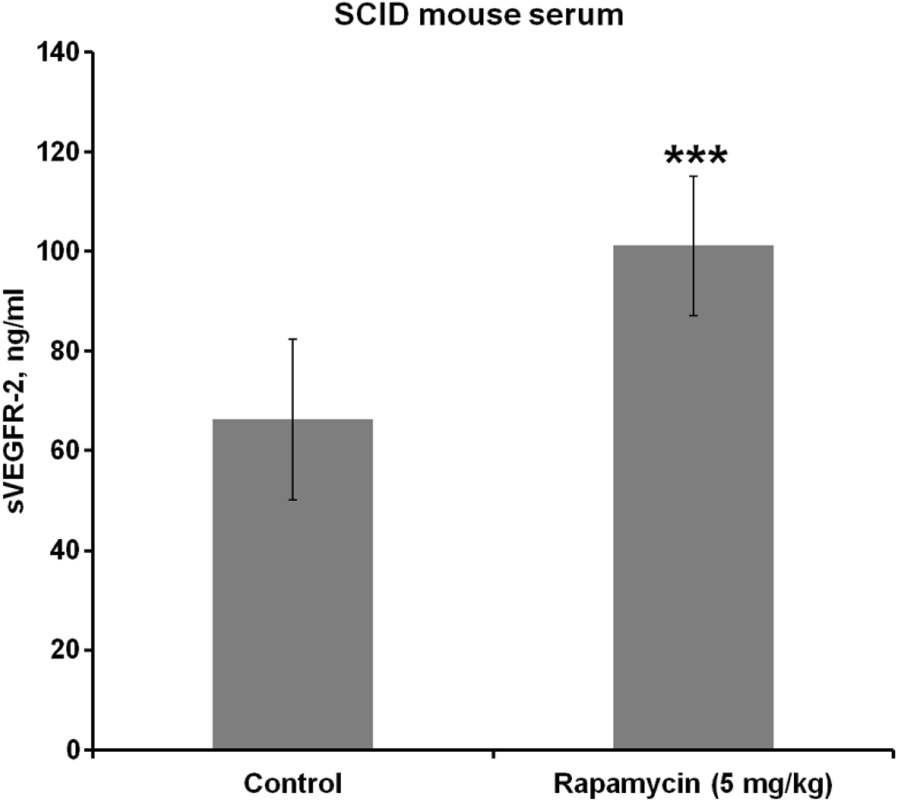
**The level of soluble VEGFR-2 was evaluated in serum of SCID mice that were sacrificed on day 21 after injection of the OSC-19 cells.** Rapamycin treatment significantly increased the level of soluble VEGFR-2 in serum of SCID mice (p = 0.0001; t-test). Serum samples from 9 control mice and 10 rapamycin-treated mice were evaluated.

Interestingly, rapamycin treatment significantly increased the level of soluble VEGFR-2 in serum of SCID mice compared to control (p = 0.0001; Figure 3).

### mTOR inhibition suppresses LEC proliferation and VEGFR-3 expression

**Figure 4:**
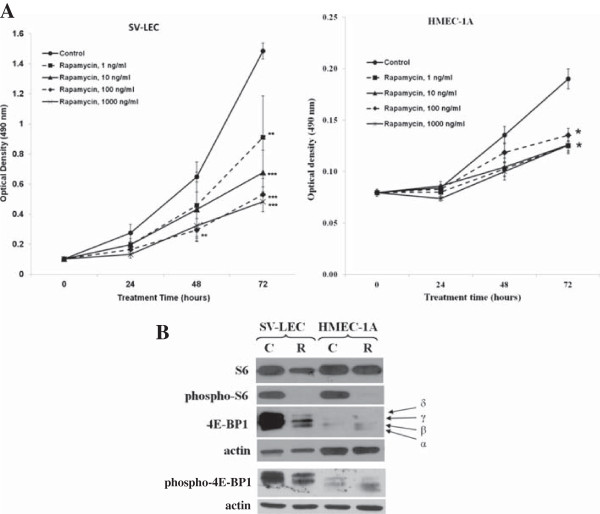
**Effects of rapamycin on growth of lymphatic endothelial cells. A**, Growth-inhibitory effects of various concentrations of rapamycin (1-1000 ng/ml) on SV-LEC and HMEC-1A cells. Optical density results are presented as means ± SD of 3 independent experiments. (* - *p* < 0.05 vs. control;** - *p* < 0.01 vs. control; one-way ANOVA with Tukey's multiple comparison post-hoc test). **B**, Western blot analysis showing an inhibition of the mTOR signaling pathway by rapamycin (100 ng/ml, 72h) in SV-LEC and HMEC-1A cells. C – Control; R – Rapamycin (100 ng/ml, 72h).

We found significant inhibition of lymphatic endothelial (LEC) proliferation in both LEC lines at all doses of mTOR inhibitors tested (1-1000 ng/ml). The growth of SV-LEC and HMVEC-1A cells were inhibited by >35% after 72 h (P < 0.05), indicating potent anti-lymphatic effects of mTOR inhibitors (Figure 4A). Interestingly after 72 h of rapamycin treatment, we noted a modest but statistically significant increase in a percentage of apoptotic cells in SV-LEC cell (control samples 6.65 ± 1.50%; rapamycin-treated samples 10.20 ± 2.46%; p = 0.0099). By comparison, there was no significant change in percentage of apoptotic cells for HMEC-1A cell line (control samples – 5.04 ± 1.39%; rapamycin-treated samples – 6.04 ± 1.99%; p > 0.05). These findings indicate a significantly higher inhibition of proliferation of SV-LEC cells than HMEC-1A cells by rapamycin.

**Figure 5:**
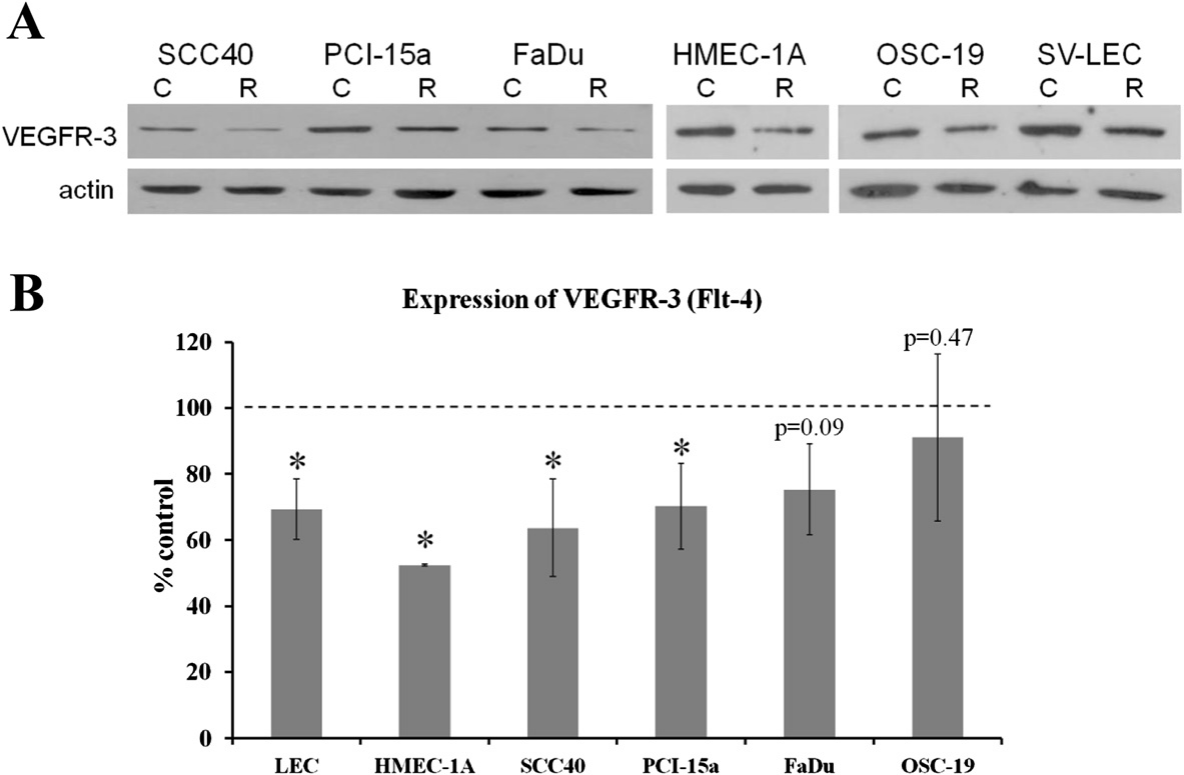
**Effects of rapamycin on VEGFR-3 expression. A**, Western blot analysis of VEGFR-3/Flt-4 expression in LECs and HNSCC cells after rapamycin treatment (100 ng/ml, 72h). **B**, Expression of pro-lymphangiogenic VEGFR-3 compared to control in the cell lines tested. The intensities of the VEGFR-3 bands were quantified at least in six independent sets of samples and statistical significance was determined using the paired two-tailed t-test.

The effects of rapamycin on mTOR signaling in LECs were evaluated by Western Blotting analysis. Inhibition of mTOR signaling was demonstrated by a significant decrease in phosphorylation of ribosomal protein S6 at Ser235/Ser236 and by a shift of the phosphorylated isoforms to non-phosphorylated “α” isoform of 4E-BP1 (Figure 4B). Interestingly, treatment with rapamycin decreased VEGFR-3 (Flt-4) expression in both LEC and HNSCC cells. We found a significant inhibition of VEGFR-3 expression after rapamycin treatment in both LEC cell lines as well as in two of four HNSCC cell lines tested, namely SCC40 and PCI-15a (Figure 5). Expression of the lymphangiogenic growth factor receptor VEGFR-3 in LEC cells, in SCC40 and PCI-15a HNSCC cells, was decreased by more than 30% after rapamycin treatment compared to vehicle-treated control (Figure 5B; *P* < 0.05; paired two-tailed t-test). Similarly in our animal experiments we observed a decrease in VEGFR-3 expression in lingual tumor tissue from 0.65 ± 0.99 in control group to 0.36 ± 0.25 in rapamycin-treated group. However due to high variability results were not significant (p = 0.177).

## Discussion

Dissemination of tumor cells to regional lymph nodes via the lymphatic system represents the first step in HNSCC metastasis and is the most important poor prognostic factor for disease recurrence. Tumor-associated lymphangiogenesis plays an active role in metastatic disease spread by providing escape routes for cancer cells and is supported by significant correlation between intratumoral lymphatic vessel density and lymph node metastasis [25,26]. HNSCC are highly vascular tumors with remarkable expansion of both blood and lymphatic vascular networks in head and neck area. In our previous study we showed an equally high density of blood and lymphatic vessels in HNSCC patients, underscoring the fact that HNSCC is not only highly angiogenic, but also highly lymphangiogenic [20]. Accumulating evidence now supports rapalogues potent activity against tumor blood vasculature and we have shown that mTOR inhibitors have potent anti-angiogenic effects in HNSCC. Temsirolimus (CCI-779) significantly suppressed angiogenesis in HNSCC xenografts, decreasing intra-tumoral microvessel density by 42% (P < 0.001) [27]. Similarly in our current study we found a significant 36% inhibition of blood microvessel density by rapamycin in the HNSCC orthotopic tumor model as well. Several studies show rapamycin also exerts anti-lymphangiogenic effects *in vitro*[28], blocks *in vivo* lymphangiogenesis in pancreatic cancer [29], and reduces regenerative lymphangiogenesis in a skin flap model [28]. Together these findings underscore the importance of mTOR-targeted therapy in inhibiting both tumor angio- and lymphangiogenesis. Unlike blood vessel angiogenesis, rapalogues effects on tumor-associated lymphangiogenesis are not well understood, but could provide critical additional target for mTOR inhibitors in the treatment of HNSCC. Recently, in the study by Gutkind et al we demonstrated anti-lymphatic properties of rapalogues in an orthotopic model of HNSCC generated by injection of UMSCC2 cells into the tongue of SCID/NOD mice [20]. In this study we obtained further evidence for the anti-lymphatic properties of mTOR inhibitors employing OSC-19 orthotopic model of HNSCC and investigated the mechanisms of rapalogues anti-lymphatic effects using *in vitro* and *in vivo* models.

Treatment of SCID mice with 5 mg/kg of rapamycin for 16 days significantly lowered lymphatic microvessel density and significantly reduced lymphovascular invasion and decreased the incidence of cervical lymph node metastasis compared to vehicle-treated controls. Furthermore, rapamycin significantly suppressed the extent of metastatic tumor cell spread within the lymph nodes. Most tumor-positive lymph nodes in the control group (78%) demonstrated complete replacement of the normal lymph node architecture with tumor cells. Conversely, the majority (74%) of positive cervical lymph nodes extracted from rapamycin-treated mice demonstrated only minimal tumor cell spread, with only few metastatic tumor cells localized to subcapsular sinuses, an early stage of cervical lymphatic metastasis known as ‘micrometastasis’. This suggests that rapamycin can delay lymphatogenous metastatic spread in head and neck cancer, potentially impeding extracapsular extension of squamous cell carcinoma nodal metastases, a significant poor prognostic factor for decreased patient survival [30].

The results obtained in the animal experiment employing an orthotopic murine model of HNSCC were further supported by *in vitro* study findings. The LEC proliferation assay showed that mouse and human lymphatic endothelial cells are highly sensitive to mTOR inhibitors, which decreases LEC proliferation by >35% in 72h of treatment. Interestingly we observed a moderate, but significant increase in apoptotic cell death after rapamycin treatment for a faster proliferating SV-LEC cell line, but not for HMEC-1A cell line, which showed only a minimal increase in the number of apoptotic cells. Potent anti-lymphatic effects of the rapalogues have now been associated with inhibition of mTOR signaling.

Not only angiogenesis, but lymphangiogenesis too plays an important role in promoting tumor growth and metastasis. The lymphatic system is a main conduit for initial metastasis for many types of solid tumors, including head and neck cancer. VEGF-C and VEGFR-3 are not only expressed by lymphatic EC, but also by a variety of HNSCC cell lines, including the HNSCC cell lines used in this study (SCC40, FaDu, PCI-15a, OSC-19) (Figure 5A). The VEGF-C/VEGFR-3 axis plays an important role in cancer progression through several cellular pathways [26]. Activation of the VEGF-C/VEGFR-3 axis in lymphatic ECs promotes lymph node metastasis, while binding of VEGF-C to VEGFR-3 creates a positive-feedback ‘autocrine loop’ which further enhances VEGF-C release, to dramatically stimulate cancer cell proliferation as well as lymphangiogenesis [26]. In our study we found that rapamycin strongly suppressed VEGFR-3 expression in both human and mouse lymphatic EC (Figure 5B). Rapalogues also significantly inhibited VEGFR-3 expression in several HNSCC cell lines. Because rapalogues down-regulate VEGFR-3 expression in lymphatic endothelial cells and some HNSCC cells it suggests mTOR inhibitors can suppress this vicious cycle of autocrine growth stimulation to decrease the number of lymph node metastasis, one of the most important factors contributing to poor head and neck cancer prognosis and survival. Mechanistically, another study coauthored by one of the authors of this paper showed that rapamycin affects VEGFR-3 protein expression in LEC cells by inhibiting protein synthesis and promoting protein degradation of VEGFR-3. Importantly rapamycin did not alter the VEGFR-3 mRNA level [31].

Another important observation from this study was that rapamycin significantly increased the level of soluble VEGFR-2 in serum samples in SCID mice implanted with HNSCC. We also observed a rapamycin-induced upregulation in the level of soluble VEGFR-2 in serum samples of nude mice with FaDu HNSCC xenograft tumors (Ekshyyan O., Moore-Medlin T., Nathan CO; unpublished observation). Recently, a soluble form of VEGFR-2 (sVEGFR-2) that is produced by alternative splicing has been identified as an endogenous selective inhibitor of lymphatic vessel growth [32,33].

In a recent study by Silver et al [33] sVEGFR-2 expression was found to be inversely correlated with lymphatic vessel density in head and neck malignant tumors. Interestingly sVEGFR-2 was not expressed in lymphatic vessels, but its expression was specific to the endothelial cells in blood vessels in both malignant tissue as well as adjacent normal tissues [34]. Furthermore it was demonstrated that gene therapy with a splicing variant esVEGFR-2 that produces soluble VEGFR-2 significantly suppresses tumor growth and lymph node metastasis in a mouse mammary cancer model [35].

Because soluble VEGFR-2 binds VEGF-C it may competitively inhibit VEGF-C-induced activation of pro-lymphangiogenic and angiogenic signaling. sVEGFR-2 release could be used as a potential biomarker of anti-lymphangiogenic and angiogenic responsiveness in clinical trials of mTOR inhibitors and warrants further investigation.

## Conclusions

Our results demonstrate that mTOR inhibitors potently inhibit lymphatic proliferation by interfering with expression of VEGFR-3, an essential lymphatic growth factor receptor necessary for LEC growth and survival. Furthermore, our data suggest that mTOR inhibitors can suppress autocrine and paracrine growth stimulation of tumor and lymphatic endothelial cells by impairing VEGF-C/VEGFR-3 axis and release of soluble VEGFR-2. In an orthotopic murine model of HNSCC rapamycin significantly suppressed lymphovascular invasion, decreased the incidence of cervical lymph node metastasis and delayed the spread of metastatic tumor cells within the lymph nodes. Our findings therefore suggest that mTOR inhibitors can effectively control lymphatogeneous metastasis, the primary predictor of poor survival in HNSCC.

## Competing interest

The authors declare that they have no conflict of interest.

## Authors’ contributions

OE contributed to project design, performed animal experiments, analyzed data, participated in data interpretation, and prepared the manuscript. TNM-M assisted in animal experiments and conducted VEGFR-2 ELISA assays. MCR, KS and MAB participated in animal experiments. XR performed the cell culture experiments and western blot analyses. FA, a study pathologist who was blinded to the study details and sample identification, performed histology evaluations. JSA contributed to project design, participated in data interpretation and edited the manuscript. CON, a project leader, envisioned the study, participated in its design, data interpretation, coordination and final manuscript preparation. All authors read and approved the final manuscript.

## Pre-publication history

The pre-publication history for this paper can be accessed here:

http://www.biomedcentral.com/1471-2407/13/320/prepub
